# Hood’s Illustration of Hydropathy

**Published:** 1849

**Authors:** 


ARTICLE VI.—*Hood's Illustration of Hydropathy.*

"It has been our good fortune, since reading Claridge on hydropathy, to see a sick drake avail himself of the "water cure," at the dispensary in Saint James' Park. First, in wading in, he took a "Fuse bad," then took a "Sitz bad," and then turning his curly tail up in the air, he took a "Kopf bad." Lastly, he rose almost upright on his latter end, and made such a flapping with his wings, that we really expected he was going to shout "Priesnitz for ever." But no such thing. He only said, "quack! quack!! quack!!!"

